# Measuring the Value of a Practical Text Mining Approach to Identify Patients With Housing Issues in the Free-Text Notes in Electronic Health Record: Findings of a Retrospective Cohort Study

**DOI:** 10.3389/fpubh.2021.697501

**Published:** 2021-08-27

**Authors:** Elham Hatef, Gurmehar Singh Deol, Masoud Rouhizadeh, Ashley Li, Katyusha Eibensteiner, Craig B. Monsen, Roman Bratslaver, Margaret Senese, Hadi Kharrazi

**Affiliations:** ^1^Center for Population Health IT, Johns Hopkins School of Public Health, Baltimore, MD, United States; ^2^The Institute for Clinical and Translational Research, Johns Hopkins School of Medicine, Baltimore, MD, United States; ^3^Department of Biomedical Engineering, Johns Hopkins Whiting School of Engineering, Baltimore, MD, United States; ^4^Atrius Health, Newton, MA, United States

**Keywords:** electronic health record, free-text, clinical notes, housing, natural language processing, social determinants of health

## Abstract

**Introduction:** Despite the growing efforts to standardize coding for social determinants of health (SDOH), they are infrequently captured in electronic health records (EHRs). Most SDOH variables are still captured in the unstructured fields (i.e., free-text) of EHRs. In this study we attempt to evaluate a practical text mining approach (i.e., advanced pattern matching techniques) in identifying phrases referring to housing issues, an important SDOH domain affecting value-based healthcare providers, using EHR of a large multispecialty medical group in the New England region, United States. To present how this approach would help the health systems to address the SDOH challenges of their patients we assess the demographic and clinical characteristics of patients with and without housing issues and briefly look into the patterns of healthcare utilization among the study population and for those with and without housing challenges.

**Methods:** We identified five categories of housing issues [i.e., homelessness current (HC), homelessness history (HH), homelessness addressed (HA), housing instability (HI), and building quality (BQ)] and developed several phrases addressing each one through collaboration with SDOH experts, consulting the literature, and reviewing existing coding standards. We developed pattern-matching algorithms (i.e., advanced regular expressions), and then applied them in the selected EHR. We assessed the text mining approach for recall (sensitivity) and precision (positive predictive value) after comparing the identified phrases with manually annotated free-text for different housing issues.

**Results:** The study dataset included EHR structured data for a total of 20,342 patients and 2,564,344 free-text clinical notes. The mean (SD) age in the study population was 75.96 (7.51). Additionally, 58.78% of the cohort were female. BQ and HI were the most frequent housing issues documented in EHR free-text notes and HH was the least frequent one. The regular expression methodology, when compared to manual annotation, had a high level of precision (positive predictive value) at phrase, note, and patient levels (96.36, 95.00, and 94.44%, respectively) across different categories of housing issues, but the recall (sensitivity) rate was relatively low (30.11, 32.20, and 41.46%, respectively).

**Conclusion:** Results of this study can be used to advance the research in this domain, to assess the potential value of EHR's free-text in identifying patients with a high risk of housing issues, to improve patient care and outcomes, and to eventually mitigate socioeconomic disparities across individuals and communities.

## Introduction

The adoption of electronic health records (EHRs) among U.S. hospitals and outpatient facilities has dramatically increased over the last decade (1, 2). Meaningful Use criteria (3, 4), the main driver of increased EHR adoption (5), has incentivized a higher capture rate of demographic and clinical information (6). Moreover, clinical informaticians and health information technology (HIT) experts have started to assess and optimize the documentation and collection of social determinants of health (SDOH) in EHRs for specific subpopulations of patients (7–12); however, SDOH documentation is still an uncommon practice in EHRs (13).

Despite the growing effort to standardize coding for SDOH concepts (14) such as Logical Observation Identifiers Names and Codes (LOINC) (15), SDOH variables are infrequently captured in EHR's structured fields and are often limited to certain SDOH types within specific clinical conditions (e.g., child abuse within the pediatric population; smoking cessation in primary care) (16, 17). However, SDOH challenges may be discussed with healthcare providers during visits and recorded in EHRs as free-text notes (i.e., providers' notes). Most SDOH variables are still captured in the unstructured fields of EHRs such as admission or clinical progress notes (14). For example, lack of social support among older adults is mentioned considerably more in geriatric notes compared to coded EHR data or other structured data sources such as insurance claims (7, 18).

While the HIT challenges exist, collecting SDOH information and implementing SDOH-specific interventions on a patient-level has become a priority for value-based care settings operating under specific organizational structures such as accountable care organizations or patient-centered medical homes (19, 20). Various factors have played a role in increasing the priority of SDOH collection among value-based settings. Some payers have started to mandate the collection of SDOH variables using survey instruments [e.g., Center for Medicare and Medicaid Innovation's Comprehensive Primary Care Plus (21) and some Medicaid (22) and private plans (23) among contracted value-based providers]. Additionally, certain states have recently introduced SDOH-derived variables to adjust the global budgets of their contracted health providers (24) [e.g., neighborhood stress index in Massachusetts' Medicaid program (20)].

Despite the incentives of value-based health systems to collect patient-level SDOH, operational challenges in rolling out large-scale SDOH surveys have limited such efforts on a population level (23, 25). Thus, the EHR free-text notes might provide a more complete or accurate accounting of SDOH challenges; however, traditional approaches for review and abstraction of patient information from medical record notes are laborious, expensive, and slow. Recent developments in text mining and natural language processing (NLP) of digitized text allow for reliable, low-cost, and rapid extraction of information from EHRs (7, 8, 18). Developing NLP algorithms that could function in different healthcare systems would improve the generalizability and application of such methods in extracting social needs from the EHR's free text. Thus, EHR text mining methods can be integrated within value-based operations to improve the identification of patient populations with SDOH challenges.

This study attempts to evaluate a practical text mining approach (i.e., advanced pattern matching techniques using regular expressions; RegEx) in identifying phrases referring to housing challenges, an important SDOH domain affecting value-based healthcare providers, using EHR of a large multispecialty medical group in New England region, United States. To present how this approach would help the health systems to address the SDOH challenges of their patients we assess the demographic and clinical characteristics of patients with and without housing issues and briefly look into the patterns of healthcare utilization among the study population and for those with and without housing challenges. The development of generalizable text mining methodologies with promising performance will help to identify social needs of patients for research purposes and to enhance the value of EHRs for population health management of at-risk patients across different health systems.

## Methods

### Data Source

We used de-identified EHR data from a large multispecialty medical group from New England, United States. We utilized data on a cohort of members who received health insurance coverage between 2011 and 2013 (based on data availability and agreement with the medical group about data access) and were assigned to this medical group as their primary source of medical care from this health plan. We extracted both structured and unstructured EHR data. Structured EHR data included age, gender, ICD-9 diagnosis codes in different settings, and the number of visits to the emergency department (ED), in-patient (IP) visits (hospitalization), or outpatient (OP) clinic visits. Unstructured data included free-text provider notes for all patients who had at least one note between the years 2011 and 2013. We did not have any limitations in selecting the provider notes and only excluded lab results and radiology and pathology reports. We explored the use of text mining techniques (i.e., pattern matching using RegEx) to determine housing challenges in the unstructured data. The institutional review board at Johns Hopkins Bloomberg School of Public Health reviewed and approved this project. Written informed consent from the participants of the study was not required by local legislation and national guidelines.

### Identifying SDOH Challenges

**Figure 1:**
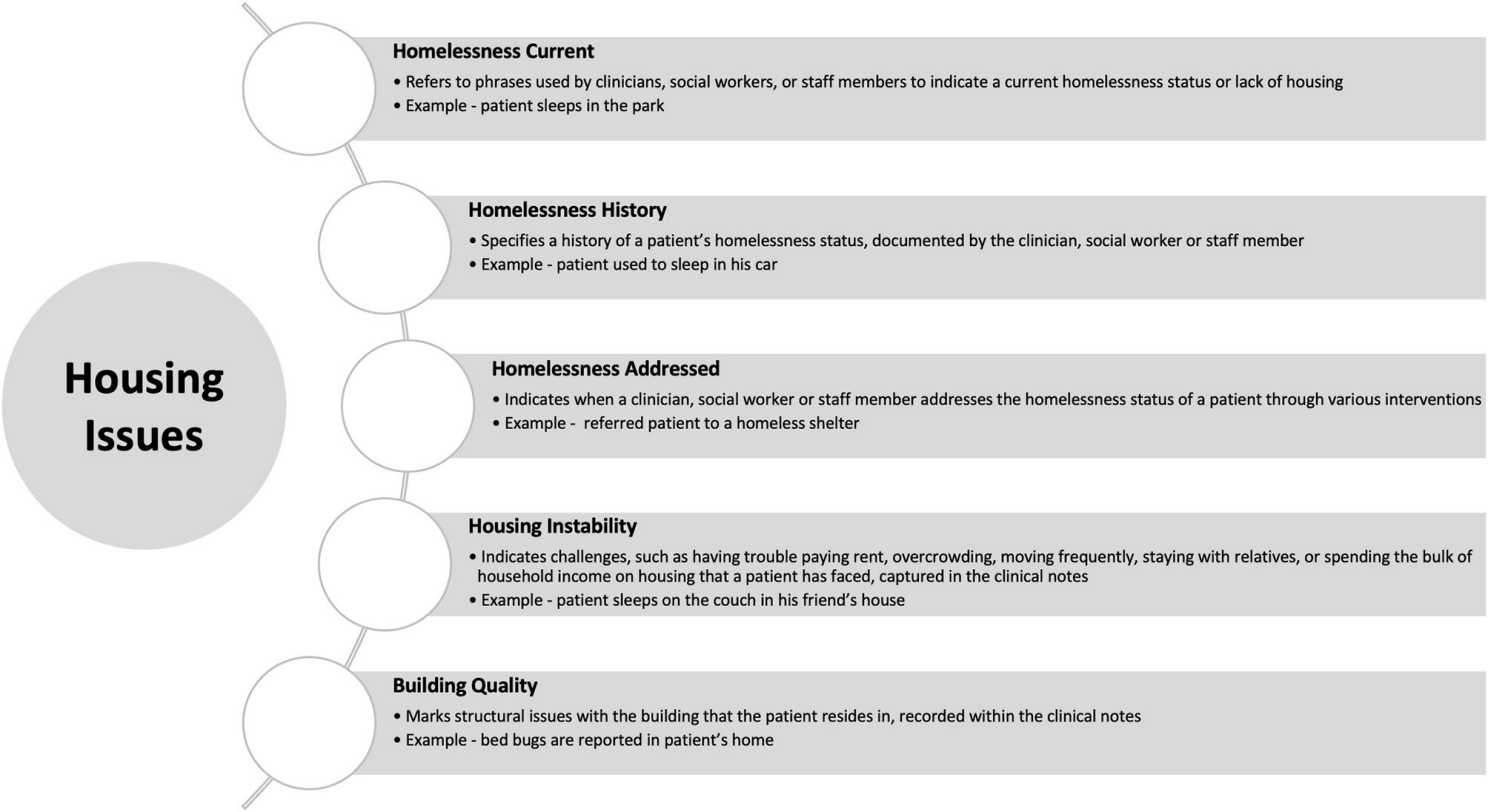
Selected categories of housing challenges, definition of each category, and type of phrases associated with each one in the electronic health record.

The data custodian identified housing issues as a growing source of challenge in their population. To address this need, the research team reviewed published articles in peer-reviewed journals, using PubMed as the preferred database. After reviewing the available evidence on housing challenges with high-impact on healthcare utilization and outcomes and consulting the subject matter experts we decided to determine five categories of housing challenges. The categories included: homelessness current (HC), homelessness history (HH), homelessness addressed (HA), housing instability (HI), and building quality (BQ). Figure 1 presents each selected category, how we defined each category, and the type of phrases associated with each one in the EHR.

Homelessness was split into three distinct categories due to different operational interventions (clinical and social) addressing each category. For example, referring a patient to a homeless shelter does not apply if the patient only has a history of homelessness but is not currently homeless. Also homelessness status of a patient may change or a patient may have two or more homelessness statuses. We addressed this issue by reporting the housing challenges at a note level, when each encounter of homelessness was counted separately, and at a patient-level when a patient was considered homeless if they had at least one encounter of homelessness. We did not report the longitudinal change in the homelessness status for each patient. The HC and HH status was linked to the specific encounter when they were documented in the EHR and were reported both at a note level and patient level.

### Generating Phrases for Each Housing Category

To identify notes containing housing issues, we used hand-crafted linguistic patterns that a team of experts developed. We first reviewed ICD-10, Current Procedural Terminology (CPT), LOINC codes, Systematized Nomenclature of Medicine (SNOMED) terminologies (14), and the description of housing issues in public health surveys and instruments [e.g., American Community Survey (26), American Housing Survey (27), The Protocol for Responding to and Assessing Patients' Assets, Risks, and Experiences (PRAPARE) (28), and the Accountable Health Communities tool from the Center for Medicare and Medicaid Innovation (21)]. We also reviewed phrases derived from a literature review of other studies and the results of a manual annotation process from a past study (7). To craft the linguistic patterns the expert team developed a comprehensive list of all available codes and specific content areas for each selected housing domain and matched them across different coding systems. Supplementary Table 1 presents examples of available codes and phrases for different categories of housing issues.

The expert team developed phrases based on aspects of the housing issues addressed in the codes, terminologies, and surveys. We further refined those phrases to address potential overlap with clinical phrases as well as learning from the underlying EHR's free-text manual tagging process. We categorized the refined phrases into green, yellow, and red phrases in multiple iterations. Green phrases indicated an active housing challenge referring to the existence of the housing issue during the encounter. Yellow phrases indicated a potential risk for a housing issue but were not conclusive. Red phrases were factors not necessarily correlated with a housing challenge. We only assessed the presence of the green phrases in free-text notes.

### Development of the Regular Expression Patterns

We intended to develop a text mining approach that could be used in a healthcare system with minimal effort and no need for advanced computational capacity, hence we used the RegEx (i.e., pattern matching) as our text mining approach. We developed multi-level RegEx patterns using green phrases for each housing category. We then developed a custom web-based application and a backend Structured Query Language (SQL) database to automate the execution of the RegEx patterns, to provide advanced RegEx functionality (e.g., negation, context detection), and for storing/preparing the results for further analysis.

### Development of the Training and Validation Dataset

The training dataset included 2,564,344 free-text clinical notes in the EHR of 20, 2017 patients. To develop the validation dataset we selected a sample of 100 patients based on the ICD-9 codes indicating a possible housing issue in their EHR structured data (20 patients for each category of the housing categories). We randomly selected 20 additional patients from the rest of the population who did not have any ICD-9 codes indicating a housing issue in their structured EHR (a total of 120 patients for the validation dataset).

Our SDOH expert (EH) trained two annotators to review and independently tag phrases describing any housing issues in the free-text EHR notes for the selected sample of 120 patients. We further customized an open-source application to pre-highlight keywords referring to housing challenges in the EHR free-text notes of the patients. The annotators initially annotated 3 test patients to assess inter-rater reliability and were consequently further trained to ensure higher agreement levels. Each annotator manually annotated all EHR records for half of the sample patients using in-house built-in functions of the customized open-source application. A third annotator then reviewed all annotated phrases for potential false positive (FP) cases across all 120 patients.

### Assessing the Performance of the Text Mining Approach

We used two different techniques to assess the performance of the RegEx text mining approach. First, we randomly selected and manually assessed 100 phrases per category of housing challenges identified by the RegEx techniques and documented the true positive (TP) and FP instances. Second, we compared the RegEx results against the manually annotated sample of 120 patients. The following sections provide more details of the two approaches.

#### Assessment #1: True Positive and False Positive Rates Among Random Patients

We first iteratively pruned the raw results of the RegEx technique to reduce potential high FP RegEx patterns. After finalizing the fine-tuning of the RegEx patterns, we extracted 100 random phrases per category of housing challenges from the pruned RegEx results and performed a phrase level assessment to calculate TP and FP rates. Supplementary Table 2 includes sample phrases found by the RegEx technique. The table lists TP findings (i.e., the RegEx found a correct housing challenge) and FPs (i.e., the RegEx found a phrase that was not a housing challenge – falsely identified as positive) for each housing category (i.e., except homeless history, as RegEx did not find any matches). Some categories did not result in 100 patients hence this assessment was limited to the maximum number of phrases identified by the RegEx pattern technique (e.g., HC only returned 65 phrases hence we assessed 65 phrases for this category). A total of 372 patients were assessed by this methodology across all housing categories. We defined precision as TP/(TP+FP), representing the positive predictive value in the text mining field. This approach did not provide false negative (FN) rates [i.e., missed recall (sensitivity) rate calculations] but offered a larger sample of patients identified by the RegEx patterns (i.e., max 100 phrases times 5 categories).

#### Assessment #2: Recall (Sensitivity) and Precision (Positive Predictive Value) of the RegEx Model

The second approach, a common evaluation approach in the text mining domain, provided both recall (sensitivity) and precision (positive predictive value) measures for the RegEx technique – as it generated TP, FP, and FN rates – but was limited to 120 sample patients whose EHR records were manually annotated for housing issues. We defined TP as cases where RegEx matched the annotators' tagging (i.e., matching the housing categories) and FP as cases where RegEx found an incorrect phrase that was not annotated by the annotators. FN included phrases that the annotators deemed relevant, but RegEx did not mark them as a housing issue. We calculated TP, FP, and FN at three levels of phrase, note, and patient. We did not use true negative (TN) cases in the assessment due to the large text not being identified or annotated by either method (i.e., RegEx or annotators). We defined recall as TP/(TP+FN) representing the sensitivity concept in the text mining domain and precision as TP/(TP+FP) representing positive predictive value. Due to the lack of TN results in the text mining field, we did not report specificity. We used the basic R function (the R version: 3.5.1) to calculate the recall (sensitivity) and precision (positive predictive value).

### Clinical Characteristics and Healthcare Utilization

We assessed the impact of housing issues on healthcare utilization including inpatient, ED, and outpatient visits. We defined (1) the inpatient visits as the acute care inpatient hospitalization stays, regardless of cause excluding pregnancy and delivery, newborns, and injury, (2) ED visits as those that were not the precursors to subsequent observation stays and inpatient hospital stays in the same period, and (3) the outpatient visits as the instances where patients received ambulatory care in outpatient settings. To describe a patient's health status, we assigned each ICD diagnosis code to one or more of 32 diagnosis groups referred to as Aggregated Diagnosis Groups (ADGs) (29) (see Supplementary Table 3 for more details) and also grouped over 8,600 diagnoses into condition categories. We also calculated the Charlson Comorbidity (30) Index, a weighted index to predict the risk of death within 1 year of hospitalization for patients with specific comorbid conditions. Additionally, we calculated the Elixhauser Comorbidity Index (31), a method of categorizing comorbidities of patients based on ICD diagnosis codes found in administrative data. The ADG, Charlson, and Elixhauser scores were used to measure the burden of chronic conditions and comorbidities in our analysis.

## Results

The study data included EHR structured data for a total of 20,342 patients and 2,564,344 free-text clinical notes. The mean age in the study population was 75.96 (SD: 7.51). Additionally, 58.78% of the cohort were female. Table 1 presents the demographic and clinical characteristics of the total study population and those with and without housing issues. Patients with housing issues were older (mean ages of 78.40, 78.78, and 77.98 years for homeless patients, and those with housing instability and building quality issues) than those with no housing issues (mean age of 75.9 years). Patients with housing issues were more female (69.60%, 70.62%, 61.11% for homeless patients, and those with housing instability and building quality issues) than those with no housing issues (58.62%).

**Table 1:** Demographic and clinical characteristics of study population categorized by housing issues^a^.

	**All patients**	**No housing issues** ^**b**^	**Homelessness** ^**b**^	**Housing instability** ^**b**^	**Building quality** ^**b**^
Patient Count	20,342	19,919	125	160	162
**Age – mean (SD)**					
	75.96 (7.51)	75.90 (7.49)	78.40 (7.86)	78.78 (7.78)	77.98 (7.95)
**Gender – female %**					
	58.78	58.62	69.6	70.62	61.11
**Comorbidity index – mean (SD)**					
Charlson^c^	1.66 (1.65)	1.64 (1.64)	2.50 (1.20)	2.69 (2.04)	2.53 (1.20)
Elixhauser^d^	3.84 (2.71)	3.81 (2.69)	5.82 (3.27)	5.91 (3.15)	5.34 (3.20)
Charlson weighted	2.47 (2.72)	2.45 (2.71)	3.56 (3.10)	3.84 (3.32)	3.61(3.13)
Elix weighted AHRQ^e^	5.21 (10.41)	5.14 (10.35)	8.81 (12.15)	8.37 (13.42)	8.74 (12.57)
Elix weighted VW^f^	5.92 (8.55)	5.85 (8.50)	9.57 (9.84)	9.36 (10.16)	9.62 (10.43)
**Utilization markers – patient count (%)**					
Emergency department	7,103 (34.92)	6,854 (34.45)	78 (62.40)	101 (63.13)	87 (53.70)
Inpatient	4,145 (20.38)	3,969 (19.95)	67 (53.60)	76 (47.50)	48 (29.63)
Outpatient	10,637 (52.29)	10,325 (51.90)	100 (80.00)	125 (78.13)	108 (66.67)

*Dx, diagnosis; SD, standard deviation*.

a *Patients with mentions of any domains of housing issues in their free-text note or those with relevant ICD-9 codes were identified as patients with housing issues*.

b *Some patients had multiple housing challenges. Therefore, the sum of figures in the columns for Homelessness, Housing Instability, and Building Quality is higher than the actual number of patients with housing challenges (“All patients – No Housing Issues” column)*.

*Due to the large sample size, the differences in the demographic and clinical characteristics between patients without housing issues (column 3) and those with different categories of housing issues (columns 4–6) were statistically significant*.

c *Charlson score is a weighted index that is predictive of the risk of death within 1 year of hospitalization for patients with specific comorbid conditions*.

d *Elixhauser score is calculated based on a method of categorizing comorbidities using diagnosis codes found in clinical data, which is predictive of hospital readmission and in-hospital mortality*.

e *A version of the Elixhauser score developed by the Agency for Healthcare Research and Quality (AHRQ) (32)*.

f *A version of the Elixhauser score developed by van Walraven et al. (33)*.

### Clinical Characteristics and Healthcare Utilization

**Figure 2:**
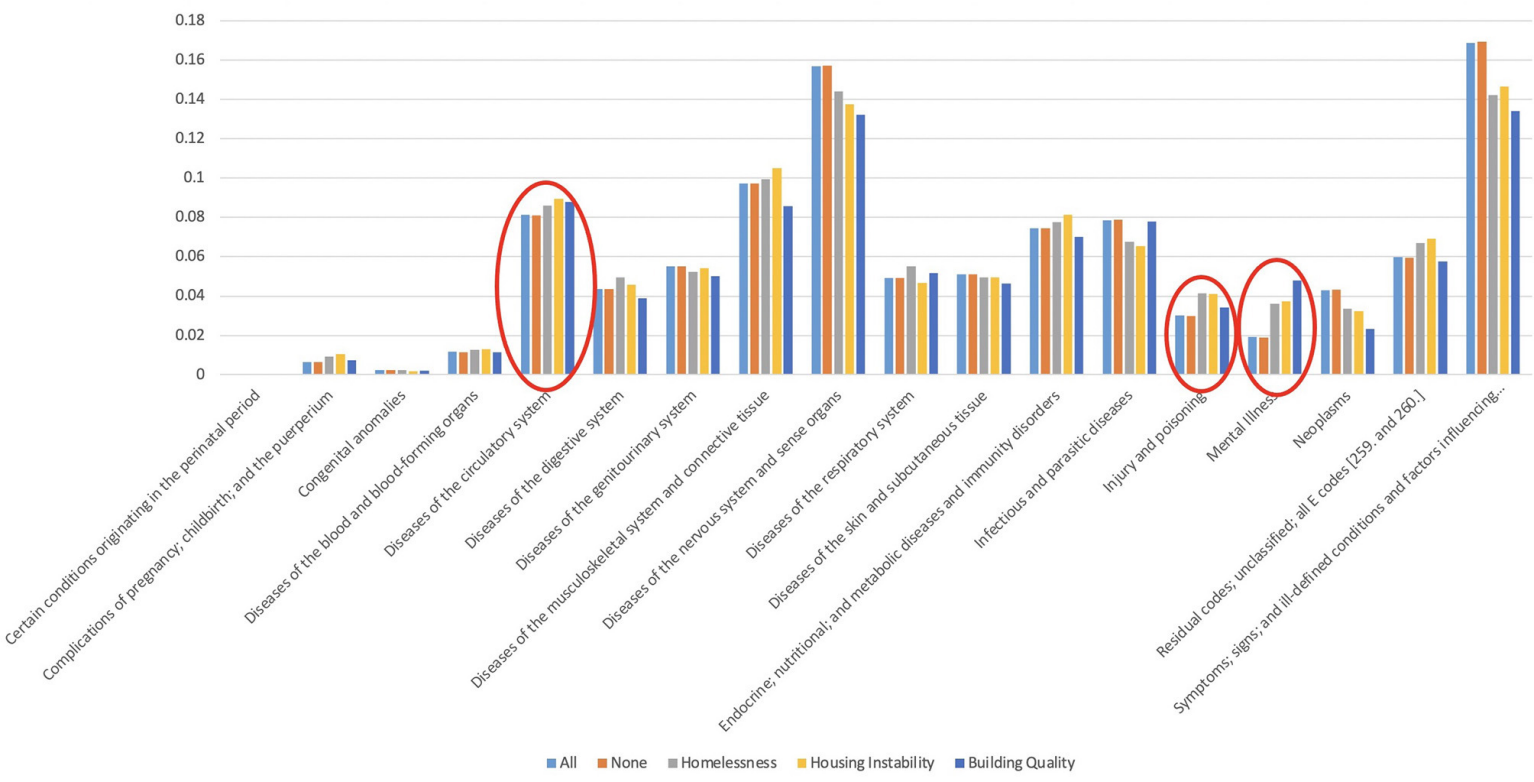
Distribution of housing issues for patients with different clinical conditions in the study population. The groups identified by the red circles have statistically significant differences.

Table 1 also presents the results of descriptive analyses for patients with housing issues, those with no housing issues, and the general population. Patients with housing issues were sicker and had higher comorbidity scores than the overall population and those with no housing issues. They also utilized the healthcare services more often. For instance, 62.40% of patients with homelessness, 63.13% of patients with housing instability, and 53.70% of patients with building quality issues had an ED visit during the study period. The ED utilization was at 34.45% for those without housing issues. Among other notable findings was the high number of outpatient visits among patients with housing issues and particularly those with homelessness (80% of patients with homelessness had outpatient visits during the study period). Figure 2 presents the distribution of housing issues for different clinical conditions. For example, a higher frequency of housing issues was noted among those with a mental illness.

### Findings of the RegEx Text Mining Technique

Table 2 depicts the total number of phrases, notes, and patients found for each housing category using the RegEx text mining methodology. The RegEx text mining identified 526 unique phrases, 494 (0.02%) unique notes, and 369 (1.82%) unique patients with housing issues. We did not define the phrase-level denominator hence phrase percentages could not be calculated. Considering the FN rate, we estimated ~890 (4.40%), unique patients, with any housing issues in our study population. Several patients had more than one housing issue documented in their free-text notes. Table 3 shows the overlap of housing categories among notes and patients. For example, 21 patients in the HA category also had other housing issues; 9 of them had housing instability and 7 had building quality issues.

**Table 2:** Total number of cases identified by the RegEx text mining technique^a^.

	**Phrases** ^**a**^		**Notes** ^**b**^		**Patients** ^**c**^		**Total patients** ^**d**^	
**Housing categories**	**No**.	**%**	**No**.	**%**	**No**.	**%**	**No**.	**%**
**Homelessness**								
Homelessness current	65	NA	60	0.002	47	0.2325	113	0.5607
Homelessness history	7	NA	7	0.000	4	0.0198	10	0.0477
Homelessness addressed	104	NA	101	0.004	76	0.3759	183	0.9066
**Housing instability**								
	176	NA	172	0.007	125	0.6183	301	1.4912
**Building quality**								
	174	NA	165	0.006	146	0.7222	352	1.7417
**Total** ^**e**^								
Unique	526	NA	494	0.019	369	1.8252	890	4.4019

a *Total number of phrases identified in the EHR during the study period describing each category of housing issues. The phrase-level denominator was not defined hence the phrase percentage could not be calculated*.

b *Total number of notes (and % of notes) in the EHR during the study period with mentions of housing challenges. The denominator included the total number of notes in the EHR during the study period*.

c *Total number of patients (and % of patients) in the EHR during the study period with mentions of housing challenges. The denominator included the total number of patients in the EHR during the study period*.

d *Total number (and % of patients) with housing issues after considering estimated false-negative rates, assuming a 41.46% recall (sensitivity) rate for patient-level RegEx analysis (see Table 4)*.

e *Unique number of phrases, notes, and patients with mentions of housing challenges in the EHR during the study period. The phrase-level denominator was not defined hence the phrase percentage could not be calculated. Some notes contained more than one housing issue and some patients reported more than one housing challenge. Therefore, the numbers are different than the sum of all categories together*.

*RegEx, regular expressions*.

**Table 3:** Total number of housing issue overlaps identified by the regex text mining technique.

	**Notes**					**Patients**				
**Category**	**HC**	**HH**	**HA**	**HI**	**BQ**	**HC**	**HH**	**HA**	**HI**	**BQ**
Homelessness current		0	2	0	0		0	5	4	4
Homelessness history	0		0	0	0	0		0	0	0
Homelessness addressed	2	0		8	1	5	0		9	7
Housing instability	0	0	8		1	4	0	9		6
Building quality	0	0	1	1		4	0	7	6	
Total number	2	0	11	9	2	13	0	21	19	17
Total %^a^	3.33	0	10.89	5.23	1.21	27.66	0	27.63	15.2	11.64

a *% of notes and patients with housing issues overlaps. The denominator is the total number of notes and patients with each category of housing issues (see Table 2 for total numbers in each category)*.

*HC, homelessness current; HH, homelessness history; HA, homeless addressed; HI, housing instability; BQ, building quality; RegEx, regular expressions*.

### Assessing Performance of RegEx Technique

Table 4 presents the results of the performance assessment of the RegEx technique using 100 randomly selected phrases (Assessment #1). Housing instability had the highest precision (positive predictive value) rate of 89%. Table 5 presents the performance assessment of the RegEx technique at the phrase, note, and patient level using manual annotation (Assessment #2). The RegEx technique had a high level of precision (positive predictive value) at all levels (96.36, 95.00, and 94.44%, respectively) but the recall (sensitivity) rate was relatively low (30.11, 32.20, and 41.46%, respectively).

**Table 4:** Performance assessment of the RegEx text mining technique using 100 random phrases.

	**Phrase level assessment**		
**Category**	**True positive** ^**a**^	**False positive** ^**a**^	**Precision (positive predictive value) %**
Homelessness current	37	28	56.92
Homelessness history	0	7	0.00
Homelessness addressed	66	34	66.00
Housing instability	89	11	89.00
Building quality	58	42	58.00

a *Number of phrases in each category of housing issues*.

*RegEx, regular expressions*.

**Table 5:** Performance assessment of the RegEx text mining technique using manual annotation.

	**Assessment level**		
**Measure**	**Phrase**	**Note**	**Patient**
True positive^a^	53	38	17
False positive^a^	2	2	1
False negative^a^	123	80	24
Recall (sensitivity) %	30.11	32.20	41.46
Precision (positive predictive value)%	96.36	95.00	94.44

a *Number of phrases, notes, and patients in each category*.

*RegEx, regular expressions*.

## Discussion

Value-based healthcare systems are increasingly at stake to address the underlying SDOH challenges of the population they serve (24). However, SDOH variables are commonly captured in EHR's free-text which makes the use of this information challenging in operational settings (14). Furthermore, healthcare providers are facing operational challenges in rolling out population-level surveys to collect individual-level SDOH information from their patients (23). Hence, text mining approaches that reveal SDOH factors within EHR's free-text can be helpful to identify patients with SDOH challenges and to implement targeted interventions for patients with such challenges.

EHR data is also gradually playing an instrumental role in the population health management efforts of value-based providers (34, 35). Compared to and in the absence of insurance claims, EHRs provide additional data types that can be utilized for risk stratification efforts (34, 36–39). EHR-derived SDOH data, such as housing challenges, can potentially help to improve these risk stratification efforts, although certain challenges such as potential immaturity of EHR's functionality across providers (40–42). SDOH data quality issues (43), and the need for complex text mining methods to extract SDOH from EHR's free-text should be addressed (7, 44). Moreover, as population health management efforts are gradually aligning clinical outcomes with public health goals (45–48), identifying SDOH factors of high-risk patients will be key in addressing underlying disparities within populations residing in states with statewide population-level global budgets such as Massachusetts (20) and Maryland (49, 50). Value-based providers may also utilize non-EHR data sources to access SDOH information (e.g., health information exchange) (51).

Therefore, the development of text mining approaches that could help extraction of SDOH information from EHR of a healthcare system regularly and could be generalizable across different healthcare systems would provide an operational solution to using this arguably largest source of SDOH information in the healthcare system. In this study, we exercised this approach by utilizing a pragmatic text-mining methodology (i.e., RegEx) and identified various phrases in EHR's free-text that reflected five categories of housing issues (i.e., three categories of homelessness, housing instability, and building quality). Our RegEx algorithm identified 369 unique patients (1.82% of the study population) with housing issues. Considering the 41.46% recall (sensitivity) of the RegEx patterns among the 120 manually annotated patients, total unique patients with housing issues after adding the estimated FNs were calculated at 890 (~4.40% of the study population). In other words, the study results showed that potentially 1 in 20 patients in our study population had a housing issue.

Furthermore, to present how this text mining approach would help the health systems to address the SDOH challenges of their patients we assessed the demographic and clinical characteristics of patients with and without housing issues and briefly look into the patterns of healthcare utilization among the study population and for those with and without housing challenges. In our study population patients with housing issues were older (mean age of ~78 years across three categories of housing issues and ~76 years among those with no housing issues), had a higher number of comorbidities (e.g., Charlson Comorbidity Index of ~2.5 across three categories of housing issues and ~1.6 among those with no housing issues), and utilized the healthcare services more often (e.g., ~54–63% ED utilization among patients with housing issues vs. ~34% among those with no housing issues). This information would help care managers, care coordinators, and social workers to tailor specific social interventions and/or conducting referrals to community-based social services organizations (52, 53). Clinicians can also utilize such information to explore the underlying housing issues at the point of care, and population health experts might use this information to better predict utilization rates associated with such patient population (54).

We provided a comprehensive approach to the performance assessment of our RegEx technique. We first assessed the performance by selecting 100 random phrases from each category of housing issues. This approach showed a precision (positive predictive value) of ~57–89% across five housing categories. We also performed manual annotation on free-text notes of 120 patients (100 patients with housing issues based on the ICD-9 codes indicating a possible housing issue in their EHR structured data, 20 patients for each category of the housing categories, and a random sample of 20 additional patients who did not have any ICD-9 codes indicating a housing issue in their structured EHR). The manual annotation revealed high precision (positive predictive value) of the RegEx technique at the phrase, note, and patient-level (~96, 95, and 94%, respectively). But the recall (sensitivity) was low at the phrase, note, and patient-level (~30, 32, and 41%, respectively). The RegEx pattern matching approach that we applied in this study is considered a basic text mining technique with rigid flexibility and potentially high FN rates. For instance, any housing phrases not embedded in the RegEx patterns will be missed in the results. The high FN rates resulted in low recall (sensitivity) for the text mining technique and the RegEx algorithm failed to identify a high number of patients with actual housing issues. However, the high precision (positive predictive value) helped to know, with high certainty, that those identified as patients with housing issues indeed were suffering from those challenges.

Manually tagging EHR's free-text for SDOH variables is an exhausting task involving several annotators spending hundreds of hours to generate the “gold standard” text. Manually annotated gold standard text is required to both assess RegEx techniques as well as train, test and evaluate advanced NLP techniques. EHR data sources that also include survey-level SDOH information will be critical in future SDOH NLP research as survey data can be treated/assumed as the gold standard text, hence enabling researchers to train, test, and evaluate the accuracy of their NLP methods. This approach might result in lower false-negative instances and improve the recall (sensitivity) of the text mining/ NLP techniques. Alternatively, approximated SDOH factors associated with the residential location/address of patients can be assessed as a proxy to train and/or validate advanced NLP techniques (e.g., compare the NLP results with SDOH variables derived based on patient's residential address).

Our results were slightly different from other studies using rule-based systems to identify social needs in free-text provider notes. For instance, Conway et al. (55) tested the performance of Moonstone, a new, highly configurable rule-based clinical NLP system for extraction of information requiring inferencing from clinical notes derived from the Veterans Health Administration. Their system achieved a precision (positive predictive value) of 0.66 (lower than ~94–96% at the phrase, note, and patient-level in our study) and a recall (sensitivity) of 0.87 (higher than ~30-41% at phrase, note, and patient-level in our study) for phrases related to homelessness and marginally housed.

In another study, Dorr et al. (56) extracted the phenotypic profiles for four key psychosocial vital signs including housing insecurity or homelessness from EHR data. They used lexical associations expanded by expert input, then, for each psychosocial vital sign, and manually reviewed the retrieved charts. Their system achieved a precision (positive predictive value) of >0.90 in all psychosocial vital signs except for social isolation. Navathe et al. (8) utilized MTERMS, an NLP system validated for identifying clinical terms within medical record text to extract social factor information from physician notes. They customized and developed the MTERMS NLP system on a randomized 500 annotated physician note training set and tested the diagnostic characteristics. After development, they validated the system by studying the diagnostic characteristics of the system vs. a gold standard manual review of a new set of randomized 600 physician notes. They achieved a precision (positive predictive value) of 1.0 and a recall (sensitivity) of 0.66 for housing instability.

While beyond the scope of this study, future efforts should also incorporate more advanced text mining approaches such as statistical NLP techniques (e.g., embedding, word2vec, and deep learning such as the recursive neural network). Recent studies utilizing such advanced NLP techniques have shown promising results in identifying syndromes not encoded properly by EHR's structured data elements (44, 57–59), Other approaches such as creating text preparation tasks may help to improve the results of the text-mining/NLP techniques. These tasks may include detecting clinical templates and repeated copy/pastes of some information in the text. They may also include detecting various sections of clinical notes that may result in the detection of false positive or false negative phrases. For instance, omitting family history of SDOH challenges and keeping the mentions of patient's specific SDOH issues may result in lower false positive or false negative instances.

This study had several limitations. First, we only identified predefined housing phrases in the EHR's free text. Second, we did not use a statistical NLP approach to assess the likelihood of notes or patients having similar phrases addressing categories of housing issues. Hence, we could not calculate the TN rates for patients and notes with housing issues. Third, we only measured the stratified rates of comorbidity and utilization among patients having any phrases related to housing issues in their free-text notes.

Moreover, we did not evaluate the net effect of the housing issues on healthcare utilization using multivariate analysis. Future research should analyze the effect of housing issues on long-term healthcare utilization while adjusting for clinical variables. The study period might also limit the study results. As in the last few years, there have been a growing number of providers and practices that actively plan for assessing and documenting the SDOH challenges in the EHR. Therefore, there might be a higher number of TP and TN instances of housing issues in the free-text EHR, which would impact the performance of the text mining techniques.

Finally, we measured the availability of housing issues regardless of the underlying socioeconomic status of the patients. Future research should expand on the underlying population denominator to patients in high need of SDOH interventions (e.g., Medicaid patients) as well as comparing NLP results with geo-driven SDOH factors (e.g., comparing the neighborhood-level housing issues, measured based on patient's residential address, with individual-level social needs found in the EHR's free-text notes).

## Conclusion

This study assessed the use of a pragmatic text mining methodology in identifying various SDOH housing factors in EHR's free text. The study results revealed a high precision (positive predictive value) for the assessed text mining approach but the recall (sensitivity) was low. The simplicity of this approach suggests its generalizability across the healthcare systems. The development of generalizable text mining methodologies with promising performance will enhance the value of EHRs to identify at-risk patients across different health systems, improve patient care and outcomes, and eventually mitigating socioeconomic disparities across individuals and communities.

## Data Availability Statement

The data analyzed in this study is subject to the following licenses/restrictions: The dataset includes patients' information in electronic health records, which are confidential to patients and their providers. Requests to access these datasets should be directed to Elham Hatef, ehatef1@jhu.edu.

## Ethics Statement

The studies involving human participants were reviewed and approved by Institutional Review Board, Johns Hopkins Bloomberg School of Public Health. Written informed consent for participation was not required for this study in accordance with the national legislation and the institutional requirements.

## Author's Note

This study attempts to evaluate a practical text mining approach (i.e., advanced pattern matching techniques) in identifying phrases referring to housing issues, an important SDOH domain affecting value-based healthcare providers, using EHR of a large multispecialty medical group in the New England region, United States.

## Author Contributions

EH supervised the development of the analysis plan, reviewed and interpreted the results, and led writing this paper. GS and MR performed the data analysis. AL, KE, CM, RB, and MS contributed to setting the overall scope and goal of the project as well as finalizing the manuscript. HK designed the overall scope and goals of the study and supervised the day-to-day operations of the project. All authors contributed significantly to the project and writing of the manuscript, reviewed the final paper, and provided comments as deemed necessary.

## Conflict of Interest

The authors declare that the research was conducted in the absence of any commercial or financial relationships that could be construed as a potential conflict of interest.

## Publisher's Note

All claims expressed in this article are solely those of the authors and do not necessarily represent those of their affiliated organizations, or those of the publisher, the editors and the reviewers. Any product that may be evaluated in this article, or claim that may be made by its manufacturer, is not guaranteed or endorsed by the publisher.
