# Supplementary material for: Measuring the Value of a Practical Text Mining Approach to Identify Patients With Housing Issues in the Free-Text Notes in Electronic Health Record: Findings of a Retrospective Cohort Study
Source: Front Public Health. 2021 Aug 27;9:697501. doi: 10.3389/fpubh.2021.697501 (PMC8429931; doi:10.3389/fpubh.2021.697501)
Supplement: Supplementary file 1 [file Table_1.DOCX]

**Table 1. Examples of Available Codes and Phrases for Categories of Housing Issues**

| **ICD10** | **SNOMED** | **LOINC** | **Public Health Surveys and Instruments** | **Literature Review** | **Manual Tagging** |  |
| --- | --- | --- | --- | --- | --- | --- |
| Homelessness (Z59.0) | Length of time homeless (442244004) | Housing status (71802-3) | Screening instruments (Housing Vital Sign & PRAPARE); What is your housing situation today? | Homeless | Living in facility |  |
| Problems related to housing & economic circumstances, unspecified (Z59.9) | Homeless (32911000) |  | PRAPARE: What is your housing situation today? I chose not to answer this question | Homelessness | Assistance with housing |  |
| Other problems related to housing & economic circumstances (Z59.8) | Homeless single person (160700001) |  | Public (section 8) housing/temporary housing/housing subsidies | No home | House will be renovated |  |
| Discord with neighbors, lodgers and landlord (Z59.2) | Homeless Family (105526001) |  | PRAPARE: What is your housing situation today? I have housing | Houseless |  |  |
| Inadequate housing (Z59.1) | Housing lack (266935003) |  | Screening instrument (Housing Vital Sign): I have housing today, but I am worried about losing housing in the future | Unhoused |  |  |
| ICD: International Classification of Diseases; LOINC: Logical Observation Identifiers Names and Codes; PRAPARE: Protocol for Responding to and Assessing Patients’ Assets, Risks, and Experiences: SNOMED: Systemized Nomenclature of Medicine. | | | | | | |

**Table 2. Categories of Housing Issues and Sample Notes for Patients with True and False Positive Phrases**

| **Category** | **Decision** | **Free-text and Sample Phrases*** |
| --- | --- | --- |
| HC | True Positive | to apply to stay in housing, and she almost **got evicted** this month. She's not eating well. Patient is having trouble getting her to work in her apartment. As a result she has been locked out of her building. 3) social: Patient's daughter has a form to apply for alert bracelet. She is interested in enrolling her mother in an assisted living, in particular House. She is waiting for patient's application for the ride to be approved. She is signing her mother of 4 adult day program this at the twice a week. |
|  |  | recent psychiatric admission for anxiety. He is **living in a shelter**, but he comes in with anxiety, has no active suicidal or homicidal ideation. He is not like his current shelter. He says it does not feel there. Please see the resident's note for past history, social history, and review of systems. |
|  |  | to hernia as it does not cover the same dermatome. **Presently is homeless** and lives at house for over one year….. to that was in a mental facility for bipolar disorder / depression per notes in chart. Pt denies having depression for which he needed to be hospitalized |
|  | False Positive | does not feel it helps but family notes that not **sleep in car** as passenger as much 2. Depression Patient reluctant to try SSRI but agreed and has been on …..x 1 Feels "same' Very tired and discouraged … |
|  |  | ……with her and her granddaughter is less accessible (**living in a shelter**). Pt agrees. Form updated. I'll ask the social worker to contact her daughter regarding options for home services and ALF. |
|  |  | He was **found sleeping in a car** and had been incontinent B&B, denies any falls. Confirmed that patient has been discharged from "he was discontinued during his rehab" and bs finger sticks 2x day. He has been incontinent since move-in, but has worsened - put on a q2 hour toileting schedule and continues needing to be changed a few times/day. Discussed with safety and elopement is a big concern. The ALF does not have a Wander guard System to help deter elopement. She has put into supervision at all times and discussed with the urgency of hiring private duty either until a bed is available in their secured dementia unit or other placement can be obtained |
| HH | True Positive | << no phrase found to be TP >> |
|  | False Positive | disorder with radiculopathy [723.4] Reports **history of displaced** disc at C7/T1 with impingement on right correlating with radiation down the right arm by MRI. ? Arthralgia of knee, right [719.46] ? Urinary retention [788.20] Two episodes requiring catheter drainage circa 20.… |
|  |  | about the same maybe even better, b/c my doorknob used to be **displaced** over and to the side, but now it's straight ahead." Pain: None Red eye: None Flashes: None Floaters: None Other symptoms… |
|  |  | Mild anterolisthesis and stenosis at L2-3. 3. **History of displacement** of lumbar intervertebral disk with compression of thecal sac at L1-2. 4. History of left-sided lumbar radicular symptoms, now resolved.… |
| HA | True Positive | **living in low income** housing, awaiting Prescription Advantage application - patient advised to contact Worker MVES Transportation concerns: No - still drives local, takes bus or The Ride Home Environment/Stressors: Much improved, was homeless Community Supports/Services: - not on service at this time. MVES - Line, MOW, Homemaker. The Ride Social Support concern: No…. |
|  |  | the floor and has trouble walking upstairs, On a **list for housing** in…. ; should improve living situation on 3rd Community Supports/Services: personal care and home making through services Social Support concern: No, nephew lives in Communication issues: No Substance abuse: No Medical issues: Medical problem concerns: YES….. |
|  |  | at (likely unrealistic) Of note-patient **lives in supportive housing** They have an on-site Social Worker. from Services Will engage as needed and as allowed by patient Will also explore with patient any other unmet needs Later same day Spoke with patient She plans on doing the prep at home… |
|  | False Positive | physical therapy as a way to improve recovery and **assess house need** for home care services. Two days, began to receive multiple calls from the patient's , who lived in , his daughter , and the daughter of Mrs. daughter , whose name is… |
|  |  | of house work stairs: floor, 1 step to get in **house services**: none supports: family, close friends difficulty affording medications/bills: no functional history: activities: keeps busy with gardening … |
|  |  | the patient needs **assessment for household** chores. 1. the patient lives with her granddaughter. who is 28 years old. has lived with pt. a few years. 2. relationship status widowed 3. type of … |
| HI | True Positive | …services but does want to talk with **social worker re housing issues**. Will forward note to , MSW, for follow up on Monday..... |
|  |  | he is in the process of moving as she **applied for affordable housing.** She will call back once she has moved and wants to come in again to be re-shown how to use the meter … |
|  |  | supplement their monthly expenses. **Need to find affordable apartment**. 3. Transportation to appointments: drives car 4. Services in the home: none at this time. Received MOW;s x 2 months but terminated this service due to not liking the food 5. Source of income: Social Security retirement/pension … |
|  | False Positive | incontinence; notes generalized arthralgias; states **difficult home situation**. Canceled colonoscopy. Wt Readings from Last 5 Encounters…. |
|  |  | … granddaughter, living with her - bipolar, **difficulty at home** with her father/ step mother: some yoga, not regular Hobby: reads… |
|  |  | rehabilitation; however, she deferred due to **issues at home** and a repeat CT scan was scheduled for her three-month interval and to follow up her x-ray. She had her chest CT scan done on… |
| BQ | True Positive | cough Contact: c/o dry cough, ? **Mold in the house**. |
|  | False Positive | causing pain in her leg. I would recommend she have a **housing unit in a flat area**. I would also recommend her unit have a laundry room on the same floor or with access to an elevator. Thank you for your help in this matter. Sincerely, M.D. RSB/dh… |
|  |  | Takes no medication for these pains and has **not used heat** or ice. Lives in a house by himself. Has someone clean. Does his own shopping and cooking and these activities do not increase pain. DIAGNOSTIC TESTS: 12/1 L ankle MRI: (does not yet know results) 1. |
|  |  | daily and which she misses every day. On, **her house was broken into** and this has caused some violation and emotional injury. She also has had some stresses recently regarding the possible sale of their house and of a condominium although she would like this, her husband has been reluctant. On examination, these rather large flat actively excoriated lesions do suggest the possibility that her self-inflicted trauma in the form of … |
| * Manually deidentified; Bolded text indicates phrases identified by the RegEx text-mining approach. BQ: Building Quality; HA: Homelessness Addressed; HC: Homelessness Current; HH: Homelessness History; HI: Housing Instability. | | |

| **Table 3. Aggregated Diagnosis Groups and Percentage of Patients in Each  Category of Housing Issues with ADG Codes** | | | | |
| --- | --- | --- | --- | --- |
| **ADG Categories** | **No SDOH** | **HL** | **HI** | **BQ** |
| ADG Code 1 : Time Limited Minor | 78.65% | 85.60% | 86.25% | 83.33% |
| ADG Code 2 : Time Limited: Minor-Primary Infections | 52.33% | 64.80% | 60.63% | 66.67% |
| ADG Code 3 : Time Limited: Major | 43.17% | 63.20% | 58.75% | 53.70% |
| ADG Code 4 : Time Limited: Major Primary Infections | 30.74% | 50.40% | 46.25% | 43.83% |
| ADG Code 5 : Allergies | 13.44% | 17.60% | 11.25% | 20.99% |
| ADG Code 6 : Asthma | 12.86% | 17.60% | 12.50% | 20.99% |
| ADG Code 7 : Like to Recur: Discrete | 77.20% | 84.00% | 87.50% | 83.95% |
| ADG Code 8 : Likely To Recur: Discrete-Infection | 44.00% | 56.80% | 56.88% | 56.79% |
| ADG Code 9 : Likely To Recur: Progressive | 25.99% | 48.80% | 48.13% | 46.30% |
| ADG Code 10 : Chronic Medical: Stable | 98.44% | 100.00% | 100.00% | 99.38% |
| ADG Code 11 : Chronic Medical: Unstable | 72.28% | 88.00% | 91.25% | 86.42% |
| ADG Code 12 : Chronic Specialty: Stable-Orthopedic | 30.18% | 32.00% | 40.00% | 41.98% |
| ADG Code 13 : Chronic Specialty: Stable-Ear, Nose Throat | 22.52% | 28.80% | 25.63% | 24.69% |
| ADG Code 14 : Chronic Specialty: Stable-Eye | 83.64% | 86.40% | 85.63% | 87.65% |
| ADG Code 15 : | 0.00% | 0.00% | 0.00% | 0.00% |
| ADG Code 16 : Chronic Specialty: Unstable-Orthopedic | 16.43% | 27.20% | 24.38% | 29.63% |
| ADG Code 17 : Chronic Specialty: Unstable-Ear, Nose, Throat | 1.49% | 3.20% | 1.25% | 1.23% |
| ADG Code 18 : Chronic Specialty: Unstable-Eye | 50.34% | 54.40% | 55.00% | 56.17% |
| ADG Code 19 : | 0.00% | 0.00% | 0.00% | 0.00% |
| ADG Code 20 : Dermatologic | 65.68% | 71.20% | 70.00% | 72.22% |
| ADG Code 21 : Injuries/Adverse Effects: Minor | 40.76% | 58.40% | 57.50% | 55.56% |
| ADG Code 22 : Injuries/Adverse Effects: Major | 43.54% | 59.20% | 68.13% | 59.88% |
| ADG Code 23 : Psychosocial: Time Limited, Minor | 24.15% | 52.00% | 46.88% | 33.33% |
| ADG Code 24 : Psychosocial: Recurrent or Persistent Stable | 36.42% | 71.20% | 67.50% | 53.70% |
| ADG Code 25 : Psychosocial: Recurrent or Persistent Unstable | 17.03% | 41.60% | 49.38% | 30.86% |
| ADG Code 26 : Signs/Symptoms: Minor | 85.80% | 95.20% | 98.13% | 97.53% |
| ADG Code 27 : Signs/Symptoms: Uncertain | 94.72% | 97.60% | 100.00% | 99.38% |
| ADG Code 28 : Signs/Symptoms: Major | 91.81% | 100.00% | 98.75% | 95.68% |
| ADG Code 29 : Discretionary | 72.48% | 78.40% | 82.50% | 75.93% |
| ADG Code 30 : See and Reassure | 57.88% | 61.60% | 55.63% | 56.79% |
| ADG Code 31 : Preventive/Administrative | 99.42% | 100.00% | 100.00% | 100.00% |
| ADG Code 32 : Malignancy | 37.37% | 44.00% | 46.25% | 37.65% |
| ADG Code 33 : Pregnancy | 11.48% | 26.40% | 20.63% | 19.14% |
| ADG Code 34 : Dental | 2.48% | 4.00% | 5.00% | 5.56% |
| ADG: Aggregated Diagnosis Groups, BQ: Building Quality, HI: Housing Instability, HL: Homelessness, SDOH: Social Determinants of Health | | | | |
